# Notes and News

**Published:** 1898-11-05

**Authors:** 


### NOTES AND NEWS.

(COLLECTED AND COMMUNICATED.)

THE Princess Louise has promised to open two wards at the West London Hospital on December 7th, at three o'clock.

A HOSPITAL has been opened at Bangkok in Siam. It is to be nursed by the sisters of a religious community, all being Europeans.

A NEW female ward is to be opened at the Campbell Hospital, Calcutta, by the Lieutenant-Governor of Bengal, in November. The ward will practically be a fair-sized hospital, as it is to accommodate 150 patients.

A SHIP has arrived at San Francisco with two persons on board suffering from the plague, whilst the captain and a sailor died from the disease on the voyage. The ship came from Hong Kong.

A DEATH from yellow fever has occurred in New York. The victim, who contracted the fever in Havana, is Colonel Waring, formerly Commissioner of the Street Cleaning Department, New York.

A NEW Infectious Diseases Hospital has been opened at Westhulme for the use of the Oldham population. The Mayor of the borough performed the ceremony. The buildings replace temporary structures, and will accommodate nearly 100 patients.

THE anti-vivisection feeling is growing very strong, and societies are being formed in many of the principal towns. The usual sensational stories of horrors perpetrated in the name of science are being circulated, and an accusation of having used orphan children for experiments has been circulated against the doctors.

RATHER a strange deadlock has occurred in relation to the opening of the Belper Infectious Hospital. It appears that the hospital is completed with the exception of a small matter entailing an expenditure of some £20. It appears that the District Rural Council are anxious the hospital should be opened, but the county medical officer will not give the necessary certificate. The estimate has been greatly exceeded, and now the Hospital Committee say they will go no further. A meeting was held respecting the matter, but nothing settled.

THE Lord Mayor of Liverpool has laid the foundation stone of a new out-patients department at the Stanley Hospital in that city. The cost, which is to be defrayed from the Roger Lyon Jones Trust Fund, is estimated at £2,500.

THE "Lewis Carroll" Cot has been formally endowed at the Children's Hospital, Great Ormond Street. It will be remembered that the friends of the Rev. C. Dodson, author of "Alice in Wonderland," commenced a subscription list to perpetuate his memory by endowing a cot, and the project has now been carried into effect.

THE National Association for the Prevention of Tuberculosis is addressing the County Council on the subject of preventing the sale of tuberculous milk and meat. Their main recommendation is that public should be substituted for private slaughter-houses. The letter sent to the County Council is signed by Sir Samuel Wilks, Sir William MacCormac, and Sir William Broadbent.

AT the last meeting of the London County Council the question of water supply of the metropolis was very fully considered. It was resolved to promote a Bill for the purchase of the water companies, and that the Bill should contain provisions for immediate connection of the various companies. Another Bill to bring additional water from Wales was also decided upon.

THE Duke of Westminster distributed the prizes to the successful students at St. George's Hospital last Saturday. In addressing the assemblage, the Duke mentioned that he was the ground landlord of the hospital. He remarked that it owned the freehold of half the site, and he urged that it should belong entirely to the hospital, and suggested that if he could meet the authorites in any way he would be pleased to do so.

A MEETING has just been held at York, called by and presided over by the Lord Mayor of that city, to consider the establishment of a consumption sanatorium for the county. Mr. H. Wharton and the Countess of Zetland were the main initiators of the movement, which was influentially supported at the well-attended meeting. After a brief speech from the Lord Mayor, Sir William Broadbent addressed the meeting, and pointed out that consumption was neither so impossible of prevention or so necessarily fatal as generally supposed. He testified to the immense importance of the open-air treatment, and suggested that the proposed institutions should be called "Homes for the Open-air Treatment of Tubercle." He urged the advantage of establishing institutions of the kind near all large centres of population, and the desirability of special local efforts. Mr. Malcolm Morris, in supporting Sir William Broadbent's remarks, urged that such institutions as were proposed should be self-supporting; they were not so much required for the very poor or the rich, but for those who could not afford to go abroad, but could pay necessary expenses in their own country. Therefore he urged that the scheme should be commenced in a tentative manner, and developed as advisable. The result of the meeting was that, it having been decided as advisable to establish a sanatorum for Yorkshire, it was agreed to constitute a committee to carry out the work, and contributions of over £2,000 were announced as promised at the meeting.

